# *N* ^ε^−Lysine Acetylation of a Bacterial Transcription Factor Inhibits Its DNA-Binding Activity

**DOI:** 10.1371/journal.pone.0015123

**Published:** 2010-12-31

**Authors:** Sandy Thao, Chien-Sheng Chen, Heng Zhu, Jorge C. Escalante-Semerena

**Affiliations:** 1 Department of Bacteriology, University of Wisconsin, Madison, Wisconsin, United States of America; 2 Department of Pharmacology and Molecular Sciences and High-Throughput Biology Center, Johns Hopkins University School of Medicine, Baltimore, Maryland, United States of America; 3 Graduate Institute of Systems Biology and Bioinformatics, National Central University, Jhongli, Taiwan; Baylor College of Medicine, United States of America

## Abstract

Evidence suggesting that eukaryotes and archaea use reversible *N* ^ε^-lysine (*N* ^ε^-Lys) acetylation to modulate gene expression has been reported, but evidence for bacterial use of *N* ^ε^-Lys acetylation for this purpose is lacking. Here, we report data in support of the notion that bacteria can control gene expression by modulating the acetylation state of transcription factors (TFs). We screened the *E. coli* proteome for substrates of the bacterial Gcn5-like protein acetyltransferase (Pat). Pat acetylated four TFs, including the RcsB global regulatory protein, which controls cell division, and capsule and flagellum biosynthesis in many bacteria. Pat acetylated residue Lys180 of RcsB, and the NAD^+^-dependent Sir2 (sirtuin)-like protein deacetylase (CobB) deacetylated acetylated RcsB (RcsB^Ac^), demonstrating that *N* ^ε^-Lys acetylation of RcsB is reversible. Analysis of RcsB^Ac^ and variant RcsB proteins carrying substitutions at Lys180 provided biochemical and physiological evidence implicating Lys180 as a critical residue for RcsB DNA-binding activity. These findings further the likelihood that reversible *N* ^ε^-Lys acetylation of transcription factors is a mode of regulation of gene expression used by all cells.

## Introduction

Post-translational modification by reversible *N* ^ε^-lysine (*N* ^ε^-Lys) acetylation of transcription factors (TFs) and transcription-related factors such as DNA-binding proteins has been reported as a means of regulating gene expression in eukaryotes [reviewed in [1], [2]] and archaea [3], [4], but not in bacteria. The probability that *N* ^ε^-Lys acetylation affects gene expression in bacteria is high for two reasons. First, Gcn5-like protein *N-* acetyltransferases (GNATs) [5] and NAD^+^-dependent Sir2-like protein deacetylases (a.k.a. sirtuins) [6], are conserved in all domains of life, and together, GNATs and sirtuins modulate the acetylation state of proteins involved in diverse cellular processes. Second, recently reported analyses of the *E. coli* proteome identified acetylated TFs, suggesting that *N* ^ε^-Lys acetylation may directly affect gene expression in bacteria [7], [8]. Supporting experimental evidence for these findings was not reported, however. Here, we provide *in vitro* evidence that reversible *N* ^ε^-Lys acetylation modulates the DNA-binding activity of a bacterial TF.

Among its many applications, proteome microarray technology [recently reviewed [9]] has been used to investigate post-translational modifications, including protein acetylation [10] and phosphorylation in yeast [11], and to study nucleic acid-protein interactions in *E. coli* [12]. Here, we used this technology to screen an *E. coli* proteome microarray (∼4,256 proteins; [12]) for substrates of the *Salmonella enterica* protein acetyltransferase (Pat) enzyme, a bacterial GNAT involved in the post-translational regulation of central metabolic enzymes [13], [14], [15].

The analysis and verification of proteome microarray data suggested that Pat acetylated several bacterial TFs. Subsequent work focused on RcsB, the response regulator of a complex signal transduction system involved in diverse processes including cell division, and capsule and flagellum synthesis [reviewed in [16], [17]]. RcsB can behave as either an activator or repressor in its regulation of target genes, and can bind DNA either as a homodimer [18] or a heterodimer with accessory cofactor RcsA [19], [20]. Together, RcsB/RcsA repress the expression of the *flhDC* genes [21], whose products positively regulate flagellum biosynthesis genes.

Here we report biochemical and LC-MS/MS data that showed RcsB was acetylated by Pat at a single Lys residue, Lys180, which resides in the DNA-binding, helix-turn-helix (HTH) motif of the protein. Acetylation was not detected after incubation of Pat-acetylated RcsB (RcsB^Ac^) with sirtuin deacetylase, CobB [13], [22], demonstrating reversibility. We isolated genetically encoded RcsB^Ac^, and show that the protein lost its ability to bind DNA. By generating substitutions at Lys180 that either abolished or mimicked acetylation, we provide *in vitro* and *in vivo* evidence that further implicate Lys180 as a critical residue for RcsB-dependent repression of the *flhDC* genes. More specifically, mutant RcsB proteins carrying substitutions at this residue were no longer acetylated by Pat, lost their ability to bind DNA, and failed to regulate gene expression *in vivo*.

## Results

### Proteome microarray experiments reveal TFs as substrates of the Pat enzyme

**Figure 1:**
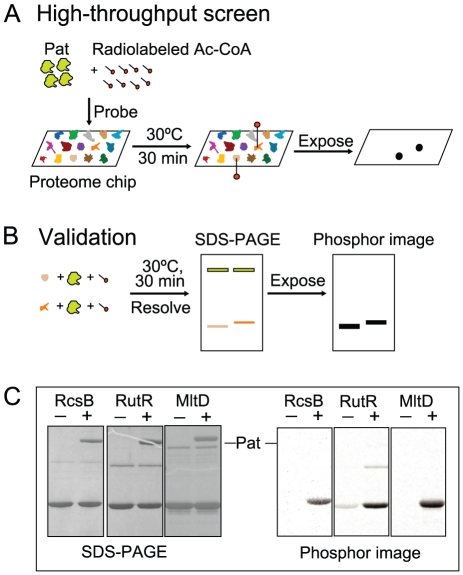
Schematic of a Pat-probed *E. coli* proteome microarray followed by analysis and subsequent validation. **A**. *High-throughput screen.* A proteome chip consisting of >4,000 *E. coli* proteins was incubated with Pat enzyme and [^14^C, C-1]-Ac-CoA. The chip was exposed to X-ray film for detection of Pat-dependent acetylation. **B**. *Validation.* Putative target proteins were individually incubated with [^14^C, C-1]-Ac-CoA in the absence or presence of Pat. The reactions were resolved by SDS-PAGE, and the prepared gels exposed to a phosphor screen for signal detection. **C**. *Representative images.* RcsB, RutR and MltD proteins were incubated with [^14^C, C-1]-Ac-CoA in the absence (- sign) or presence (+ sign) of Pat. Left panels show denaturing polyacrylamide gels of each reaction mixture, while the right panels show the corresponding phosphor images.

To identify proteins that could be modified by the *S. enterica* protein acetyltranferase (Pat) enzyme, we incubated [^14^C, C-1]-acetyl-Coenzyme A (Ac-CoA) and Pat with an *E. coli* proteome microarray [12], and compared the results to a control experiment performed in parallel in the absence of Pat. Twenty-nine putative protein substrates were identified (Table S1). To validate the microarray data, we scaled up the purification of the putative protein substrates using plasmids from the ASKA library of *E. coli* ORFs [23], and purified proteins were individually incubated with Pat and [^14^C, C-1]-Ac-CoA. A schematic of the method is presented (Fig. 1A,B) along with representative results (Fig. 1C). A list of proteins confirmed to be substrates of Pat is also provided (Table S1). We validated Pat-dependent acetylation of seven proteins: MltD, RpsD, RutR, McbR, RcsB, YcjR and YbaB; four of these are reported TFs, namely, RpsD [24], McbR [25], RcsB [26], and RutR [27]. To date, *N* ^ε^-Lys acetylation of these proteins has not been reported.

### RcsB^Ac^ is deacetylated by CobB, an NAD^+^-dependent sirtuin deacetylase

**Figure 2:**
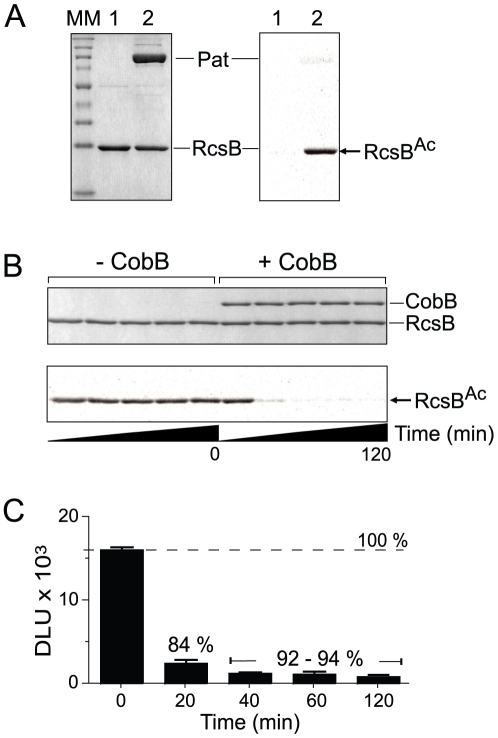
RcsB is a substrate of the Pat acetyltransferase and CobB sirtuin deacetylase enzymes. **A**. Un-tagged RcsB was incubated with [^14^C, C-1]-Ac-CoA without (*lane 1*) and with Pat (*lane 2*), analyzed with SDS-PAGE (left panel), and exposed to a phosphor screen for detection of radioactivity (right panel). **B**. Radiolabeled RcsB^Ac^ was incubated with NAD^+^ and CobB sirtuin deacetylase. Samples were removed over time after the addition of CobB protein. The upper panel shows the SDS-PA gel, while the lower panel shows the corresponding phosphor image. **C**. Quantification of the amount of label removed by CobB from radiolabeled RcsB^Ac^. Percentages are relative to the label associated with RcsB^Ac^ in a reaction devoid of CobB. Each determination is the average of duplicate reactions. MM, molecular mass markers; DLU, digital light units.

The CobB sirtuin is the deacetylase that, together with Pat, controls the acylation state of several metabolic proteins [14], [15], [22], [28]. To determine whether RcsB^Ac^ was a substrate of CobB, we incubated Pat-acetylated RcsB with CobB and NAD^+^. The results revealed that RcsB^Ac^ was a substrate of CobB, with up to 92% removal of the acetyl moiety from RcsB^Ac^ within 40 min under the conditions used (Fig. 2A–C).

### Pat acetylates residue Lys180 in the DNA-binding motif of the RcsB response regulator

**Figure 3:**
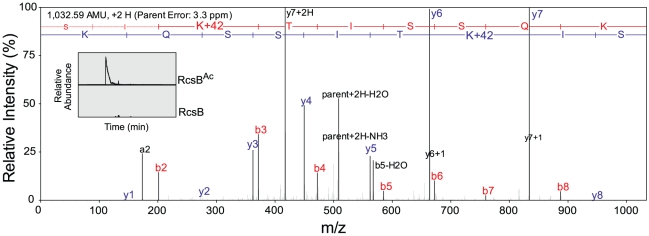
Identification of acetylation site K180^Ac^ on peptide SIK^Ac^TISSQK by nanoLC-MS/MS from trypsin digests of RcsB. For peptide fragmentation analysis, raw MS/MS data were converted to Mascot generic format (mgf) using Trans Proteomic Pipeline (Seattle Proteome Center, Seattle, WA), and the resulting mgf files were used to search NCBI non-redundant *Proteobacteria* amino acid sequence database using an in-house Mascot search engine (Matrix Science) with lysine acetylation, methionine oxidation and cysteine carbamidomethylation as variable modifications. Raw MS/MS spectrum for *m/z*  = 517.2998 Da was unambiguously assigned to peptide SIK^Ac^TISSQK. The observed continous amino-terminal b ions and carboxy-terminal y ions resulting from the dissociation of the peptide backbone map the acetylation site to Lys180. Inset picture is of extracted chromatograms for the *m/z*  = 517.2998 Da of RcsB^Ac^ and un-acetylated RcsB (RcsB) protein samples. Chromatogram x- and y-axis units are equally scaled; x-axis is in Time (min), and y-axis units are in Relative Abundance (0–100%).

NanoLC-MS/MS analysis of tryptic peptides representing 85% sequence coverage of RcsB^Ac^ unambiguously identified a single residue, Lys180, as the site modified by Pat (Fig. 3). Residue Lys180 is of interest because it is located within the predicted DNA-binding motif of *E. coli* RcsB. In spite of its location and positive charge, Lys180 in *Erwinia amylovora* RcsB has not been reported to make significant contacts with DNA [29]. Nevertheless, corresponding Lys residues in the DNA-recognition motif of other members of the LuxR-type family of transcription factors have been shown [*e.g.* Lys 179 of DosR [30]] or predicted [*e.g.* Lys179 of SsrB [31] and Lys41 of GerE, [32]] to make direct contacts with DNA.

### Acetylation and substitutions at Lys180 cause a defect in binding of RcsB to a *flhDC* promoter DNA probe

**Figure 4:**
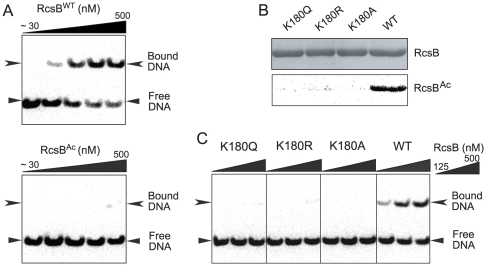
Acetylation of Lys180 inhibits RcsB DNA-binding activity, mimicking the effect of substitutions that block interactions at this position. Reactions of RcsB proteins with the *flhDC* operator sequence contained equimolar RcsA, and were analyzed by EMSAs. **A.** The DNA-binding activities of wild-type RcsB (RcsB^WT^, top panel) and acetylated RcsB (RcsB^Ac^, bottom panel) were assessed. RcsB protein concentrations from left to right: 31.25, 62.5, 125, 250, 500 nM. **B.** Wild-type and variant RcsB proteins were incubated with Pat and [^14^C, C-1]-Ac-CoA, resolved on a denaturing polyacrylamide gel (top), and exposed to a phosphor screen (bottom). **C.** Assessment of the DNA-binding activities of wild-type and variant RcsB proteins. RcsB protein concentrations from left to right: 125, 250, 500 nM.

We hypothesized that modifications or substitutions at Lys180 would have a negative effect on the DNA-binding activity of RcsB. To investigate this possibility, we performed electrophoretic mobility shift assays (EMSAs) using conditions similar to those reported elsewhere [21]. A DNA probe incubated with genetically encoded RcsB^Ac^ protein [obtained as described by Neumann, *et al.* [33], [34]; Fig. S1] lost the ability to bind DNA (Fig. 4A, bottom panel), as compared to wild-type RcsB protein (Fig. 4A, top panel). Further, single-amino acid substitutions at Lys180 (i.e. RcsB^K180A^, RcsB^K180R^, RcsB^K180Q^) resulted in variant proteins that had two features. One, they were not acetylated by Pat (Fig. 4B), and two, they lost DNA-binding activity as compared to wild-type RcsB tested under the same conditions (Fig. 4C). The effect of the K180Q substitution was of note since Gln substitutions have been reported to mimic *N* ^ε^ *-*Lys^Ac^ [35], [36].

Since *S. enterica* sv. Typhimurium LT2 Pat and CobB enzymes were used in proteome microarray assays and subsequent experiments, we verified that the homologous enzymes in *E. coli* K-12 MG1655 also used RcsB as substrate (Fig. S2A, B). Likewise, *E. coli* Pat (annotated as YfiQ), also failed to acetylate variant RcsB proteins (Fig. S2C) under conditions similar to those used with the Pat enzyme, suggesting that Pat and YfiQ modify RcsB only once, at Lys180. The CobB, Pat, and RcsB proteins in these bacteria are 91%, 92%, and 99% identical, respectively. Because the *pat* designation has been taken for putrescine aminotransferase in *E. coli* K-12, we will continue to refer to the *E. coli* gene as *yfiQ*. Results from the above experiments provided confidence for subsequent experiments in *E. coli*.

### Substitutions of Lys180 block RcsB-dependent repression of the *flhDC* genes

The observed loss of DNA-binding activity of RcsB (Fig. 4A,C) suggested that reversible *N* ^ε^-Lys acetylation of residue Lys180 might work as a means of modulating RcsB-dependent gene expression. From the literature, we knew the Rcs system was affected by mutations in various genes, by overexpression of additional genes, or in response to environmental signals [37], [38], [39]. In *E. coli*, overproduction of RcsB from multicopy plasmids represses *flhDC* expression, with the concomitant decrease in motility [21], and activates capsule synthesis (*cps*), which results in mucoidy [26]. Overproduction of RcsB is believed to mimic the over-activation of the Rcs signal transduction system.

RcsB-dependent regulation of the *flhDC* and *cps* genes has been studied *in vitro* and *in vivo* [19], [21], [26]. High-level synthesis of capsular polysaccharide is inhibitory for *E. coli* growth, and cells have been reported to accumulate second-site mutations [26]. Because of this problem, we chose to focus on the effects from *rcsB* in multicopy in the context of flagellar synthesis.

**Figure 5:**
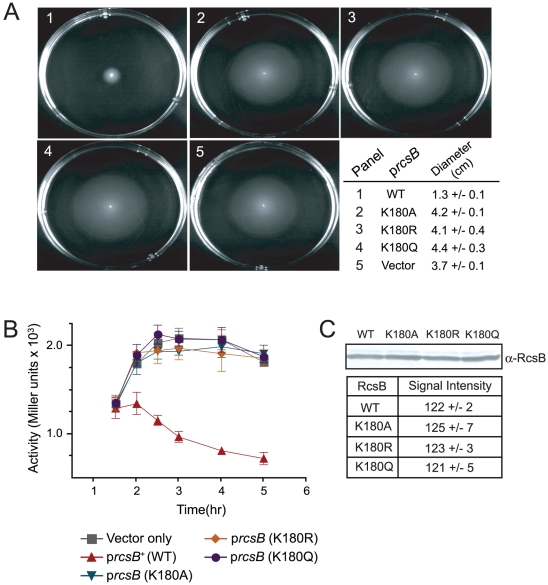
Substitutions of Lys180 de-repress expression of flagellum biosynthesis genes. **A**. Arabinose-inducible plasmids containing wild-type and mutant *rcsB* alleles were introduced into *E. coli*. The resulting strains were grown to an OD_600_∼0.8, and inoculated with a sterile needle into the center of motility plates containing arabinose (0.5% or 33 mM). The distance traveled by cells was measured after an 24-hr incubation period at 28°C, and is presented as mean ± s.d. determined from three independent experiments from individual cultures done in triplicate for each strain. **B**. The plasmids were introduced into a *rcsB* strain harboring a λ lysogen containing a P*_flhD_*::*lacZ^+^* promoter fusion, and expression of each plasmid-borne *rcsB* allele was induced with arabinose (0.5% or 33 mM). β-Galactosidase activity was measured along different stages of growth; a vector-only control was included. The data presented is the average of three independent experiments from individual cultures performed in triplicate for each strain.

To explore the effects of substitutions at Lys180 *in vivo*, we introduced into the cell wild-type and mutant *rcsB* alleles in multicopy, and determined whether the over-production of the encoded proteins would repress *flhDC* expression. Since variant RcsB proteins lost DNA-binding activity (Fig. 4C), we predicted that variant RcsB proteins would not repress *flhDC* expression *in vivo*. We cloned wild-type and mutant *rcsB* alleles under the control of arabinose-inducible promoters [40], introduced the plasmids individually into an Δ*rcsB* strain, and assessed motility. As previously described, expression of the *rcsB* ^+^ allele repressed motility [21], in contrast, expression of the mutant *rcsB* alleles did not (Fig. 5A). Plasmids and strains used in these experiments are listed in table S2 and table S3, respectively.

To quantify the effect of substitutions at Lys180 on the expression of *flhDC*, we introduced plasmids that directed the synthesis of variant RcsB proteins into an Δ*rcsB flhDC^+^* strain harboring a λ lysogen containing a P*_flhDC_-lacZ* fusion ([41], Table S3). As expected, wild-type RcsB negatively regulated *flhDC* expression [21] (Fig. 5B, triangles), while variant RcsB proteins did not (Fig. 5B). Western blot analysis showed that variant RcsB proteins were stable (Fig. 5C), ruling out the possibility the observed lack of *flhDC* repression was due to absence of RcsB.

## Discussion

Our experiments revealed that both *E. coli* and *Salmonella* YfiQ/Pat enzymes modify the bacterial response regulator, RcsB, by acetylation. Likewise, the CobB sirtuin deacetylase from both bacteria modify RcsB^Ac^ by deacetylation. The site of acetylation, Lys180, appears to be critical for RcsB DNA-binding activity, as evidenced by a lack of shift in the mobility of the *flhDC* probe when incubated with RcsB carrying acetylation or substitutions at this position. Although Lys180 of *Erwinia amylovora* RcsB was not reported to make significant contacts with a DNA fragment representing the RcsA/B box [29], Lys180 is in the middle of sequence RSIK^180^TIS, which is proposed to be the DNA-binding HTH motif in *E. amylovora* RcsB [29]. This motif is conserved in *E. coli* RcsB, and because of its location, it is likely that acetylation at Lys180 disrupts direct interactions between *E.coli* RcsB and the *flhDC* promoter. Because of its positive charge, the molecular mechanism behind the observed loss in DNA-binding that resulted from acetylation at Lys180 is probably due to neutralization of its charge, which would disrupt or hinder direct interactions with the negatively charged phosphate backbone of DNA. This has been reported for other TFs whose DNA-binding activity was attenuated by acetylation, *e.g.* the mammalian TF Foxo1 [42], a member of the FOXO family of forkhead TFs.

Although RcsB-dependent regulation of genes has been investigated for the last two decades, mechanistic details of RcsB binding to DNA as a homodimer, heterodimer or in combination with accessory factors are unclear. Also missing are the mechanistic details of the effect of phosphorylation on RcsB oligomerization and/or DNA binding.

In our hands, the affinity of RcsB for the *flhDC* operator sequence was substantially higher than the one reported in the literature (Fig. 4A). Our data show that as low as 62 nM RcsB can exert a quantifiable effect on DNA mobility. To explain this discrepancy, we note that *in vitro* approaches to studying RcsB function (*e.g.* EMSAs, DNAse I protection assays, transcription assays) have been performed with tagged RcsB variants (maltose binding protein (MBP), His-tags) [18], [19], [20], [21], [29], [43], [44]. The use of tags is likely due to the inherent difficulty in isolating transcription factors, whose concentrations are kept low. However, the use of tag technology may be problematic since, in experiments aimed at describing RcsB binding to the *flhDC* operator sequence, others reported that RcsB binding to DNA was not detected unless the His-tagged RcsB concentration in the reaction mixture reached ≥1.5 µM [21].

In fact, a constitutively active form of RcsB containing a mutation at the proposed site of phosphorylation [45], His-tagged RcsB^D56E^, was used instead for both EMSA and DNAse I protection assays, and data reported suggested that this form was more active than wild-type RcsB [21].

Likewise, others reported having to use the His-tagged RcsB^D56E^ variant to see protection from DNAse I digestion at another promoter, *osmCp1*, since neither His-tagged RcsB nor crude extract enriched in RcsB resulted in protection [46]. These authors also reported that even the His-tagged RcsB^D56E^ variant was unable to produce a band shift in EMSA experiments. This observation was attributed to possibly an unstable protein-DNA complex unable to withstand electrophoresis. Indeed, the RcsB-DNA complex was reported to be unstable, and the role of RcsA proposed to stabilize this interaction [20], [29]. The RcsA/B dimer is also likely unstable since Wehland *et al.* [20] reported no detection of dimer formation from yeast two-hybrid screening and from affinity chromatography with immobilized His-tagged RcsB.

Although RcsB has not been reported to be acetylated *in vivo*, our findings suggest that reversible *N* ^ε^ *-*Lys acetylation may be involved in regulating *E. coli* cell motility (Fig. 5A,B). This observation is not unprecedented. Recent reports showed that acetylation of the *E. coli* response regulator CheY had a negative effect on binding to its targets [47], and that CobB was able to regulate chemotaxis by deacetylation of CheY, shown *in vitro* and *in vivo* [48]. In addition, proteins directly involved in motility, MotB and Crp, have also been reported to be acetylated [8]. However, the effect of these modifications remains unclear. Further, a recent report on the regulation of the *cobB* and *pat* genes in *S. enterica* showed expression to be growth rate-dependent, and evidence showed the proteins encoded by these two genes were responsible for the reversible *N* ^ε^ *-*Lys acetylation of central metabolic enzymes in this bacterium [15]. These data suggest the possibility that other protein substrates of CobB and Pat/YfiQ may be directly or indirectly involved in processes that affect motility besides CheY and RcsB.

## Materials and Methods

### Fabrication of *E. coli* proteome chips and acetylation assay

A protein microarray containing most of the *E. coli* K-12 MG1655 proteome was prepared as described [12]. Each protein was spotted in duplicate and calf histones H3 and H4 were used as landmarks and positive controls. A description of the proteome chip acetylation assay is available in the supporting nformation Text S1 file on the PLos One website, www.plosone.org.

### Construction of the *pat*, *rcsB* and *rcsA* overexpression plasmids used to generate *N*-terminally tagged, TEV-cleavable proteins

The 2661-bp *pat (*formerly *yfiQ)* gene of *Salmonella enterica* sv. Typhimurium LT2 was PCR-amplified using 5′ and 3′ primers that included *Kpn*I and *Hind*III sites, respectively. PCR products cut with *Kpn*I and *Hin*dIII were ligated into pTEV plasmid pKLD66 [49], cut with same enzymes. Plasmid pKLD66 directs the synthesis of the protein of interest with an *N*-terminal hexahistidine-maltose-binding protein (His_6_-MBP) tag cleavable with tobacco etch virus (TEV) protease [50], [51]. The presence of the insert was verified by restriction enzyme analysis and DNA sequencing using BigDye® Terminator v3.1 protocols (Applied Biosystems). Sequencing reactions were resolved and analyzed at the University of Wisconsin Biotechnology Center. The resulting 9.3-kb plasmid was named pPAT8. The 651-bp *rcsB* and 624-bp *rcsA* genes were amplified from *E. coli* K12 MG1655, and the plasmids were constructed as described above for pPAT8. The 7.3-kb *rcsB* plasmid was named pRCSB6, the 7.0-kb *rcsA* plasmid was named pRCSA1.

### Construction of plasmids overexpressing mutant alleles of *rcsB*

Plasmid pRCSB6 was subjected to site-directed mutagenesis using the QuikChange XL kit (Stratagene) to produce variants RcsB^K180A^ (AAA to GCG; plasmid pRCSB19), RcsB^K180R^ (AAA to CGT; plasmid pRCSB20), and RcsB^K180Q^ (AAA to CAG; plasmid pRCSB10). Table S2 lists primers used to generate the *rcsB* alleles.

### Construction of arabinose-inducible plasmids for expression of *rcsB* alleles

The *rcsB* gene was amplified from *E. coli* K12 MG1655 using 5′ and 3′ primers containing terminal *Eco*RI (along with 30 nt 5′ of the start codon) and *Xba*I restriction sites, respectively. PCR products were cut with *Eco*RI and *Xba*I, and were ligated into plasmid pBAD30 [40] cut with same enzymes. The presence of the insert was confirmed by DNA sequencing. The 5.6-kb *rcsB* plasmid was named pRCSB3. Cloning of mutant *rcsB* alleles encoding variant RcsB^K180A^, RcsB^K180R^ and RcsB^K180Q^ proteins was performed as described above (Table S2).

### Overproduction of Pat, CobB, RcsB and RcsA proteins

*S. enterica* CobB protein was purified as described [14] except that plasmid pCOBB33 encoding the *cobB^+^* gene (laboratory collection) was used. Pat, RcsB and RcsA proteins were produced from plasmids (described above), which direct the synthesis of protein with ether an N-terminal His_6_-maltose-binding protein (MBP) tag or a C-terminal His_6_-tag removable by tobacco etch virus (TEV) protease cleavage [50], [51]. A two-step histidine affinity column purification method (described below) was used to purify Pat and RcsB. RcsA was purified using just the first step since the tag was needed for stability [29], [44]. Because RcsA is degraded by Lon protease [52], we overproduced His_6_-MBP-RcsA in the Lon-deficient strain ER2566.

Overexpression plasmids were transformed into strain *E. coli* C41(DE3) *yfiQ*::*kan* ^+^ (laboratory collection), and overnight cultures sub-cultured 1∶100 into 2 L of LB containing ampicillin (150 µg/ml). Cultures were grown at 37°C with shaking to an OD_600_ of 0.6, induced with IPTG (1 mM), and shaken overnight at 15°C. Cells were harvested by centrifugation, and re-suspended in 20 ml of binding buffer [20 mM sodium phosphate at pH 7.5, containing NaCl (500 mM), and imidazole (20 mM)] containing lysozyme (1 mg/ml), DNAse I (25 µg/ml) and PMSF (0.5 mM). Cells were lysed by French press (2X), and clarified cell lysate was obtained after centrifugation and filtration. Samples were loaded onto a 1-ml HisTrap HP column attached to an AKTA FPLC system (GE Healthcare).

His_7_-TEV protease (hereafter referred as rTEV protease) was purified as described [51]. rTEV protease was added to the tagged protein at a ratio of 1∶100 protease-to-protein, the mixture was incubated at room temperature for 3 hr, then dialyzed at 4°C against 20 mM sodium phosphate at pH 7.5, containing NaCl (500 mM) and TCEP (0.5 mM). An elution consisting of a linear gradient with imidazole allowed for separation of tagged and un-tagged protein. Untagged proteins were stored in HEPES buffer (50 mM, pH 7.5) containing NaCl (150 mM) and glycerol (2.7 mM), flash-frozen in liquid nitrogen and kept at −80°C.

### Construction of plasmids for overexpression of the wild-type and mutant allele of *rcsB* for the overproduction of C-terminally tagged, TEV-cleavable proteins for EMSA analysis

To generate a homogenously acetylated RcsB construct for analysis with EMSAs, we used a two-plasmid system described by Neumann, *et al.* [33], [34]. This system allows for the site-specific incorporation of *N* ^ε^-acetyllysine by way of an *Methanosarcina barkeri* acetyl-lysyl-tRNA synthetase/tRNA_CUA_ pair that responds to the amber codon. To avoid the isolation of truncated forms of RcsB^Ac^ (the construct above was N-terminally tagged), we cloned wild-type *rcsB* into pET-23a(+) (EMD) which produces a C-terminal His_6_-tagged construct. By using a 5′ primer incorporating an NdeI site and a 3′ primer incorporating an XhoI site in addition to a TEV cleavage site (5′ – CTC GAG ACC TTG GAA GTA GAG ATT CTC GTC TTT ATC TGC CGG ACT TAA – 3′) we produced a homogenous pool of RcsB protein that retained six primer derived residues ENLYFQ following rTEV cleavage. This plasmid was named pRCSB22. By incorporating an amber codon at Lys180 (AAA to TAG by site-directed mutagenesis), we produced plasmid pRCSB23 that encodes for a homogenous pool of RcsB^Ac^ (Fig. S1). Both proteins were produced and purified as described above, except *E. coli* C41(DE3) was used for expression, and cells were induced at an OD_600_ of 0.6 with 0.5 mM IPTG. Further, the amber construct was overexpressed in media, LB + spectinomycin (50 µg/ml) + kanamycin (50 µg/ml) + ampicillin (150 µg/ml), in addition to 2 mM *N* ^ε^ *-*acetyllysine (Sigma-Aldrich) + 20 mM nicotinamide at the time of induction, similar to that described [33], . Proteins encoded by these plasmids were used to assess binding activities by EMSA analysis.

### Purification of proteins from the *E. coli* ASKA library

Of the 29 putative Pat substrates identified by the microarray experiments, 22 were isolated using the ASKA collection [23].

Plasmids from the latter were transformed into strain *E. coli* C41(DE3) *yfiQ*::*kan* ^+^. Protein isolation was performed in small-scale (5 ml) or large-scale (1 L) cultures in LB containing chloramphenicol (34 µg/ml), using a Maxwell 16 System (Promega) or an FPLC system, respectively. Proteins were stored under conditions similar to those used for Pat protein unless higher salt (300 mM NaCl) and/or DTT (1 mM) were needed for stability.

### Pat-dependent acetylation of RcsB proteins

Conditions optimized for acetylation of un-tagged, wild-type RcsB were used to determine whether untagged, variant RcsB proteins were substrates of Pat. Reactions were performed in duplicate. Reactions (20 µl) contained Pat (2 µM), RcsB protein (5 µM), [^14^C, C-1]-Ac-CoA (25 µM), and TCEP (0.5 mM). Reactions were incubated at 37°C for 2 hr, followed by quenching with 4 µl 6X SDS-PAGE loading buffer and heating at 95°C for 2 min. 12-µl of reaction (50 pmol of RcsB protein) was resolved in a 12% SDS-PA gel, dried, and phosphor image obtained after 15-min exposure using a storage phosphor screen and a Typhoon Trio Variable Mode Imager and ImageQuant v5.2 software (GE Healthcare).

### Preparation and analysis of His_6_-RcsB^Ac^ by nanoLC-MS/MS analysis

Details pertaining to NanoLC-MS/MS analysis of peptides of His_6_-RcsB^Ac^ are available in the Text S1 file.

### Sirtuin-dependent deacetylation of RcsB^Ac^

The ASKA His_6_-Pat protein was used to facilitate its removal by HisMag beads (Novagen) from the acetylation reaction. Un-tagged, radiolabeled RcsB^Ac^ was prepared as described above. The CobB deacetylation assay has been described [13]. Deacetylation reactions (20 µl) contained CobB (0.8 µM), radiolabeled RcsB^Ac^ (2 µM), NAD^+^ (1 mM), and TCEP (0.5 mM) in HEPES buffer (50 mM, pH 7.5). Reactions were performed in duplicate, including a no-enzyme control. A 10-µl sample (20 pmol of RcsB) from each reaction was loaded onto a 12% SDS-PA gel. Images were obtained as described above.

### Electrophoretic mobility shift assays (EMSAs)

The LightShift Chemiluminescent EMSA Kit (Pierce) was used for binding assays. A 5′-biotinylated probe encompassing the −85 to +34 nt relative to the *flhDC* transcription start point [21], was generated by PCR-amplification from *E. coli* K-12 MG1655. Reaction volumes were 20 µl consisting of 20 fmol biotinylated-*flhD* probe and equimolar RcsB to RcsA un-tagged wild-type or variant protein in 1X Binding buffer with 50 ng/µl Poly(dI•dC). Reactions were incubated at 28°C for 30 min, 5 µl of 5X Loading Buffer was added, and 5 fmol of probe was resolved on 15-well 6% native polyacrylamide gel. Detection of chemiluminescence and image digitization were obtained from scanning on a Typhoon Trio Variable Mode Imager.

### β-galactosidase activity assays

β-Galactosidase activities were determined as described [53]. Three independent overnight cultures were grown per strain in LB containing ampicillin (150 µg/ml), sub-cultured (1∶100) into 10 ml of LB containing ampicillin (150 µg/ml) and arabinose (0.5% or 33 mM) in borosilicate tubes. Cell density was monitored shortly after inoculation into stationary phase. Cultures were incubated at 37°C with shaking. At each time point, 80 µl of culture was removed and enzyme activity measured. Refer to Table S3 for strain information.

### Swimming motility assays

Refer to Table S3 for strain information. For investigation of the Lys180 substitutions on the function of RcsB, three independent overnight cultures of each strain were grown in LB containing ampicillin (150 µg/ml). Swim plates contained tryptone (10 g/L), NaCl (5 g/L), Bacto agar (Difco; 0.25% w/v), ampicillin (150 µg/ml) and arabinose (0.5% or 33 mM), and were made immediately prior to use. Inoculation was performed using a sterile needle to puncture the middle of the agar plate. Plates were incubated at 28°C for 24 hr. The diameter of the zone of swimming was measured and photographed using a Fotodyne digital imaging system.

### Antibody preparation and Western blot analysis

Un-tagged, RcsB protein was used to elicit rabbit polyclonal antibodies (Harlan). To determine the level of wild-type and mutant RcsB proteins produced from expression of these *rcsB* alleles under the control of an arabinose-inducible promoter, cells from 10-ml cultures in LB plus ampicillin (150 µg/µl) and arabinose (0.5% or 33 mM) were harvested at a cell density of OD_600_ of 0.6 by centrifugation, then re-suspended in 0.5 ml of HEPES buffer (50 mM, pH 7.5) containing lysozyme (1 mg/ml), DNAse I (25 µg/ml) and PMSF (0.5 mM). Cells were lysed by sonication for two 1-min intervals using a Heat Systems-Ultrasonics sonicator (Model W-10) at setting 3. Cell debris was removed by centrifugation and 10 µl of supernatant was resolved in a 12% SDS-PA gel. Binding of α-RcsB antibodies to blots was visualized using alkaline phosphatase-conjugated goat α-rabbit immunoglobulin G (ThermoFisher) and NBT/BCIP chemistry. Band intensity was measured by densitometry analysis using a Fotodyne Digital Imaging system and TotalLab v2005 software. The experiment was performed in duplicate from two independent cultures.

## Supporting Information

Text S1 This file provides details of the methodology used to identify Pat substrates using an *E. coli* proteome chip, the validation procedures and results for all putative Pat substrates identified by the proteome chip labeling studies, the preparation of His6-RcsBAc for LC/MS/MS analysis, and the conditions for the mass spectrometry analysis.
(DOC)

Figure S1 **Native-PAGE, SDS-PAGE and Western blot analysis of wild-type RcsB and site-specific acetylated RcsB.** By utilizing an acetyl-lysyl-tRNA synthetase/tRNA_CUA_ pair that incorporates *N* ^ε^ *-*acetyllysine in response to the amber codon, we generated a homogenously acetylated RcsB^Ac^ construct. **A**. 500 ng of each protein was resolved on a 12% native polyacrylamide gel. RcsB^Ac^ (lane 2) runs faster than wild type RcsB (lane 1). This is because neutralization of the lysine residue increases overall negative charge density, which causes the protein to migrate faster. **B**. Western blot analysis of 500 ng of RcsB^WT^ and RcsB^Ac^, (lane 1 and 2, respectively). Polyclonal rabbit α-acetylated lysine antibodies (1:1,500; Calbiochem) show that the RcsB^WT^ and RcsB^Ac^ constructs are unacetylated and acetylated, respectively. Alkaline phosphatase conjugated secondary antibodies and NBT/BCIP chemistry was utilized for visualization.
(EPS)

Figure S2 **Verification of the acetyltransferase and deacetylase activities of *E. coli* and *S. enterica* Pat and CobB enzymes.** Purified proteins from either *E. coli* (*Ec*) or *S. enterica* (*Se*) were assayed with wild-type *E. coli* RcsB and [^14^C, C-1]Ac-CoA to verify activities. The *E. coli* enzymes were isolated from the ASKA library. **A**. Reactions were performed in duplicate. Reactions (20 µl) contained Pat (2 µM), RcsB protein (5 µM), [^14^C, C-1]-Ac-CoA (25 µM), and TCEP (0.5 mM). Reactions were incubated at 37°C for 2 hr, followed by quenching with 4 µl 6X SDS-PAGE loading buffer and heating at 95°C for 2 min. A 12-µl sample (50 pmol of RcsB protein) from each quenched reaction was resolved in a 12% SDS-PA gel, and phosphor images were obtained and analyzed using a Typhoon Trio Variable Mode Imager and ImageQuant v5.2 software (GE Healthcare). **B**. Quantification of the amount of label removed by CobB from Pat-radiolabeled RcsB^Ac^. Radiolabeled RcsB^Ac^ was incubated with NAD^+^ and CobB sirtuin deacetylase. Reactions (20 µl) contained CobB (0.8 µM), RcsB^Ac^ protein (2.0 µM), NAD^+^ (1 mM), and TCEP (0.5 mM). Reactions were incubated at 37°C for 20 min, followed by quenching with 4 µl 6X SDS-PAGE loading buffer and heating at 95°C for 2 min. A 12-µl sample (20 pmol of RcsB protein) from each quenched reaction was resolved in a 12% SDS-PA gel, and phosphor images were obtained. Each determination is the average of duplicate reactions. Percentages are relative to the label associated with RcsB^Ac^ in a reaction devoid of CobB. DLU, digital light units. **C**. Wild-type and variant RcsB proteins were incubated with *Ec*Pat and radiolabeled Ac-CoA, resolved on a denaturing polyacrylamide gel, and exposed to a phosphor screen (represented).
(EPS)

Table S1 **Proteome chip assay results and verification.** ^a^Strains contain additional mutations associated with strain VH1000 = *lacI lacZ pyrE* ^+^. The VH1000 = *lacI lacZ pyrE* ^+^ φ(*flhD-lacZ*) strain [5] was a gift from R. Gourse (University of Wisconsin-Madison). The strain is derived from *E. coli* K-12 MG1655. ^b^The *araC771*::*kan* ^+^ and *rcsB770*::*kan* ^+^ alleles were obtained from the Keio collection of in-frame deletions in *E. coli* K-12 BW25113 strain containing a deletion in the arabinose utilization genes, Δ*araBAD567* [6]. The insertion in *araC* was excised as described [7] and the *rcsB770*::*kan* ^+^ allele was introduced by phage P1-mediated transduction.
(DOC)

Table S2 ***rcsB* plasmids and primers.** ^a^Primers used to introduce the amino acid substitution. Nucleotide changes are underscored. ^b^Plasmids derived from cloning vector pBAD30 [3] for *in vivo* analysis. ^c^Plasmids derived from pTEV cloning vector pKLD66 [4] for overproduction and purification of products.
(DOC)

Table S3 ***E. coli* K-12 strains used in this study^a^.** ^a^Strains contain additional mutations associated with strain VH1000 = *lacI lacZ pyrE* ^+^. The VH1000 = *lacI lacZ pyrE* ^+^ φ(*flhD-lacZ*) strain [5] was a gift from R. Gourse (University of Wisconsin-Madison). The strain is derived from *E. coli* K-12 MG1655.
(DOC)
