# Supplementary material for: N ε−Lysine Acetylation of a Bacterial Transcription Factor Inhibits Its DNA-Binding Activity
Source: PLoS One. 2010 Dec 31;5(12):e15123. doi: 10.1371/journal.pone.0015123 (PMC3013089; doi:10.1371/journal.pone.0015123)
Supplement: Text S1 — This file provides details of the methodology used to identify Pat substrates using an E. coli proteome chip, the validation procedures and results for all putative Pat substrates identified by the proteome chip labeling studies, the preparation of His6-RcsBAc for LC/MS/MS analysis, and the conditions for the mass spectrometry analysis. (DOC) [file pone.0015123.s001.doc]

# SUPPORTING INFORMATION

***N*ε−Lysine Acetylation of a Bacterial Transcription Factor Inhibits its**

**DNA-Binding Activity**

**Sandy Thao^1^, Chien-Sheng Chen^2,3^, Heng Zhu^2^ and Jorge C. Escalante-Semerena^1*^**

^1^Department of Bacteriology, University of Wisconsin, Madison, WI 53706, USA.

^2^Department of Pharmacology and Molecular Sciences & High-Throughput Biology Center, Johns Hopkins University School of Medicine, Baltimore, Maryland 21205, USA.

^3^Graduate Institute of Systems Biology and Bioinformatics, National Central University, Jhongli 32001, Taiwan.

*Send Correspondence to: 1550 Linden Dr, Madison, WI 53706-1521; Tel: 608-262-7379; Fax: 608-265-7909; E-mail: [escalante@bact.wisc.edu](mailto:escalante@bact.wisc.edu)

**SUPPORTING EXPERIMENTAL PROCEDURES**

***E. coli* proteome acetylation chip assays.** *E. coli* proteome microarrays were blocked in Superblock Blocking Buffer (Pierce) for 1 hr, rinsed twice with binding buffer [50 mM Tris-HCl, pH 7.5 containing glycerol (0.68 mM), EDTA (0.1 mM), DTT (1 mM), sodium butyrate (10 mM), PMSF (1 mM), and KCl (50 mM)] and [^14^C, C-1]Ac-CoA (Amersham, 0.83 mCi/ml). Microarrays were probed (30 min, 30ºC) with or without 3 mg/ml of Pat enzyme in binding buffer, washed 3x for 15 min with 50 mM NaHCO_3_-Na_2_CO_3_ (3:1) buffer at pH 9.3, and centrifuged at 1,819 x *g* until dry prior to exposure to Kodak X-ray film. Acetylated proteins were visualized after 30 days. Autoradiographs were scanned, images were saved as TIFF files, and analyzed using GenePix Pro 6.0 software to determine relative acetylation levels. A list of putative targets generated according to signal strength is presented in Table S1.

**Validation of Pat protein substrates.** Proteins identified using the chip assay were isolated and individually tested using *in vitro* acetylation conditions described [1]. Acetylation assays (30 µl) contained Pat enzyme (1 µM), protein substrate (10 µM), [^14^C, C-1]Ac-CoA (20 µM; Moravek) and TCEP (1 mM) in HEPES buffer (50 mM, pH 7.5). Duplicate reactions were performed, including a no-enzyme control. Reactions were incubated at 37°C for 30 min, then quenched with 6 µl 6X SDS-PAGE loading buffer and heated at 95°C for 2 min. A 12-µl sample (100 pmol of Pat protein substrate) from each quenched reaction was resolved by 12% SDS-PAGE and visualized by Coomassie Brilliant Blue staining. Gels were dried, exposed to a phosphor screen (PerkinElmer) for 24 - 48 hr, and images were generated and analyzed using a Typhoon Trio Variable Mode Imager, and ImageQuant v5.2 software (GE Healthcare). Acetylation was considered Pat-dependent if the signal for a given protein accounted for > 50% of the total radioactivity detected, i.e., the sum of the signals for no-enzyme and plus enzyme reaction (Table S1).

**Preparation of His_6_-RcsB^Ac^ for nanoLC-MS/MS analysis.** Components of an acetylation reaction performed with non-radioactive Ac-CoA was resolved and visualized by SDS-PAGE and Coomassie Blue staining, and a band correlating with His_6_-RcsB protein was excised for analysis. A control reaction devoid of Pat enzyme was included. In-gel tryptic digestion of the His_6_-RcsB^Ac^ protein and mass spectrometric analysis of the peptides was performed at the University of Wisconsin-Madison Mass Spectrometry Facility. Tryptic digestion and peptide recovery was performed as outlined on website: <http://www.biotech.wisc.edu/ServicesResearch/MassSpec/ingel.htm>.

**NanoLC-MS/MS.** Peptides were analyzed by nanoLC-MS/MS using the Agilent 1100 nanoflow system connected to a hybrid linear ion trap-orbitrap mass spectrometer LTQ-Orbitrap (Thermo Fisher Scientific) equipped with a nanoelectrospray ion source. Capillary HPLC was performed using an in-house fabricated column with integrated electrospray emitter [2] except that 360 µm x 75 µm fused silica tubing was used. The column was packed with 5 µm C18 particles (Column Engineering) to ~ 12 cm. Sample loading (8 µl) and desalting were achieved using a trapping column (Zorbax 300SB-C18, 5 µM, 5 x 0.3 mm, Agilent) in line with the autosampler. HPLC solvents were as follows: *Loading*: 1% (v/v) ACN, 0.1 M acetic acid; *A*: 0.1 M acetic acid in water, and *B*: 95% (v/v) acetonitrile, 0.1 M acetic acid in water. Sample loading and desalting were done at 10 µl/min with the loading solvent delivered from an isocratic pump. Gradient elution was performed at 200 nL/min and increasing %B in *A* of 0 to 40 in 100 min, 40 to 60 in 15 min, and 60 to 100 in 5 min. The LTQ-Orbitrap was set to acquire MS/MS spectra in data-dependent mode as follows: MS survey scans from m/z 300 to 2000 were collected in profile mode at a resolving power of 100,000. MS/MS spectra were collected on the three most-abundant signals in each survey scan. Dynamic exclusion was employed to increase dynamic range and maximize peptide identifications. This feature excluded precursors up to 0.55 *m/z* below and 1.05 *m/z* above previously selected precursors. Precursors remained on the exclusion list for 15 s. Singly charged ions and ions for which the charge state could not be assigned were rejected from consideration for MS/MS.

**SUPPORTING INFORMATION LEGENDS**

**Figure S1. Native-PAGE, SDS-PAGE and Western blot analysis of wild-type RcsB and site-specific acetylated RcsB.** By utilizing an acetyl-lysyl-tRNA synthetase/tRNA_CUA_ pair that incorporates *N*ε*-*acetyllysine in response to the amber codon, we generated a homogenously acetylated RcsB^Ac^ construct. **A.** 500 ng of each protein was resolved on a 12% native polyacrylamide gel. RcsB^Ac^ (lane 2) runs faster than wild type RcsB (lane 1). This is because neutralization of the lysine residue increases overall negative charge density, which causes the protein to migrate faster. **B.** Western blot analysis of 500 ng of RcsB^WT^ and RcsB^Ac^, (lane 1 and 2, respectively). Polyclonal rabbit α-acetylated lysine antibodies (1:1,500; Calbiochem) show that the RcsB^WT^ and RcsB^Ac^ constructs are unacetylated and acetylated, respectively. Alkaline phosphatase conjugated secondary antibodies and NBT/BCIP chemistry was utilized for visualization.

**Figure S2. Verification of the acetyltransferase and deacetylase activities of *E. coli* and *S. enterica* Pat and CobB enzymes.** Purified proteins from either *E. coli* (*Ec*) or *S. enterica* (*Se*) were assayed with wild-type *E. coli* RcsB and [^14^C, C-1]Ac-CoA to verify activities. The *E. coli* enzymes were isolated from the ASKA library. **A.** Reactions were performed in duplicate. Reactions (20 µl) contained Pat (2 µM), RcsB protein (5 µM), [^14^C, C-1]-Ac-CoA (25 µM), and TCEP (0.5 mM). Reactions were incubated at 37°C for 2 hr, followed by quenching with 4 µl 6X SDS-PAGE loading buffer and heating at 95°C for 2 min. A 12-µl sample (50 pmol of RcsB protein) from each quenched reaction was resolved in a 12% SDS-PA gel, and phosphor images were obtained and analyzed using a Typhoon Trio Variable Mode Imager and ImageQuant v5.2 software (GE Healthcare). **B.** Quantification of the amount of label removed by CobB from Pat-radiolabeled RcsB^Ac^. Radiolabeled RcsB^Ac^ was incubated with NAD^+^ and CobB sirtuin deacetylase. Reactions (20 µl) contained CobB (0.8 µM), RcsB^Ac^ protein (2.0 µM), NAD^+^ (1 mM), and TCEP (0.5 mM). Reactions were incubated at 37°C for 20 min, followed by quenching with 4 µl 6X SDS-PAGE loading buffer and heating at 95°C for 2 min. A 12-µl sample (20 pmol of RcsB protein) from each quenched reaction was resolved in a 12% SDS-PA gel, and phosphor images were obtained. Each determination is the average of duplicate reactions. Percentages are relative to the label associated with RcsB^Ac^ in a reaction devoid of CobB. DLU, digital light units. **C.** Wild-type and variant RcsB proteins were incubated with *Ec*Pat and radiolabeled Ac-CoA, resolved on a denaturing polyacrylamide gel, and exposed to a phosphor screen (represented).

**Table S1**. **Proteome chip assay results and verification.** ^a^Strains contain additional mutations associated with strain VH1000 = *lacI lacZ pyrE*^+^. The VH1000 = *lacI lacZ pyrE*^+^ φ(*flhD-lacZ*) strain [5] was a gift from R. Gourse (University of Wisconsin-Madison). The strain is derived from *E. coli* K-12 MG1655.

^b^The *araC771*::*kan*^+^ and *rcsB770*::*kan*^+^ alleles were obtained from the Keio collection of in-frame deletions in *E. coli* K-12 BW25113 strain containing a deletion in the arabinose utilization genes, Δ*araBAD567* [6]. The insertion in *araC* was excised as described [7] and the *rcsB770*::*kan*^+^ allele was introduced by phage P1-mediated transduction.

**Table S2. *rcsB* plasmids and primers.** ^a^Primers used to introduce the amino acid substitution. Nucleotide changes are underscored.

^b^Plasmids derived from cloning vector pBAD30 [3] for *in vivo* analysis.

^c^Plasmids derived from pTEV cloning vector pKLD66 [4] for overproduction and purification of products.

**Table S3. *E. coli* K-12 strains used in this study^a^.** ^a^Strains contain additional mutations associated with strain VH1000 = *lacI lacZ pyrE*^+^. The VH1000 = *lacI lacZ pyrE*^+^ φ(*flhD-lacZ*) strain [5] was a gift from R. Gourse (University of Wisconsin-Madison). The strain is derived from *E. coli* K-12 MG1655.

^b^The *araC771*::*kan*^+^ and *rcsB770*::*kan*^+^ alleles were obtained from the Keio collection of in-frame deletions in *E. coli* K-12 BW25113 strain containing a deletion in the arabinose utilization genes, Δ*araBAD567* [6]. The insertion in *araC* was excised as described [7] and the *rcsB770*::*kan*^+^ allele was introduced by phage P1-mediated transduction.

**SUPPORTING REFERENCES**

1. Starai VJ, Escalante-Semerena JC (2004) Identification of the protein acetyltransferase (Pat) enzyme that acetylates acetyl-CoA synthetase in *Salmonella enterica*. J Mol Biol 340: 1005-1012.

2. Martin SE, Shabanowitz J, Hunt DF, Marto JA (2000) Subfemtomole MS and MS/MS peptide sequence analysis using nano-HPLC micro-ESI fourier transform ion cyclotron resonance mass spectrometry. Anal Chem 72: 4266-4274.

3. Guzman LM, Belin D, Carson MJ, Beckwith J (1995) Tight regulation, modulation, and high-level expression by vectors containing the arabinose PBAD promoter. J Bacteriol 177: 4121-4130.

4. Rocco CJ, Dennison KL, Klenchin VA, Rayment I, Escalante-Semerena JC (2008) Construction and use of new cloning vectors for the rapid isolation of recombinant proteins from *Escherichia coli*. Plasmid 59: 231-237.

5. Lemke JJ, Durfee T, Gourse RL (2009) DksA and ppGpp directly regulate transcription of the Escherichia coli flagellar cascade. Mol Microbiol 74: 1368-1379.

6. Baba T, Ara T, Hasegawa M, Takai Y, Okumura Y, et al. (2006) Construction of *Escherichia coli* K-12 in-frame, single-gene knockout mutants: the Keio collection. Mol Syst Biol 2: 2006 0008.

7. Datsenko KA, Wanner BL (2000) One-step inactivation of chromosomal genes in *Escherichia coli* K-12 using PCR products. Proc Natl Acad Sci USA 97: 6640-6645.
