# Supplementary material for: N ε−Lysine Acetylation of a Bacterial Transcription Factor Inhibits Its DNA-Binding Activity
Source: PLoS One. 2010 Dec 31;5(12):e15123. doi: 10.1371/journal.pone.0015123 (PMC3013089; doi:10.1371/journal.pone.0015123)
Supplement: Table S1 — Proteome chip assay results and verification. aStrains contain additional mutations associated with strain VH1000 = lacI lacZ pyrE +. The VH1000 = lacI lacZ pyrE + φ(flhD-lacZ) strain [5] was a gift from R. Gourse (University of Wisconsin-Madison). The strain is derived from E. coli K-12 MG1655. bThe araC771::kan + and rcsB770::kan + alleles were obtained from the Keio collection of in-frame deletions in E. coli K-12 BW25113 strain containing a deletion in the arabinose utilization genes, ΔaraBAD567 [6]. The insertion in araC was excised as described [7] and the rcsB770::kan + allele was introduced by phage P1-mediated transduction. (DOC) [file pone.0015123.s004.doc]

**Table S1. Proteome chip assay results and verification.**

|  | **Name** | **Isolated^a^** | **Pat-acetylated^b^** | **Signal (%)^c^** |
| --- | --- | --- | --- | --- |
| 1 | RpsD | + | + | 75.1 ± 1.0 |
| 2 | FlgJ | N | NT |  |
| 3 | YdgH | N | NT |  |
| 4 | YpfH | + | N |  |
| 5 | MltD | + | + | 85.5 ± 2.3 |
| 6 | YkgN | N | NT |  |
| 7 | YgiF | + | N |  |
| 8 | WcaA | N | NT |  |
| 9 | RfbX | N | NT |  |
| 10 | RluC | + | N |  |
| 11 | GlxR | + | N |  |
| 12 | YcjR | + | + | 60.6 ± 1.7 |
| 13 | YecA | + | N |  |
| 14 | McbR | + | + | 69.7 ± 0.1 |
| 15 | YbaB | + | + | 56.6 ± 0.7 |
| 16 | Sun | + | N |  |
| 17 | PurB | + | N |  |
| 18 | RcsB | + | + | 65.2 ± 2.0 |
| 19 | Gst | + | N |  |
| 20 | CheZ | + | N |  |
| 21 | LigB | N | NT |  |
| 22 | YggD | + | N |  |
| 23 | Tdh | + | N |  |
| 24 | PhoW | N | NT |  |
| 25 | AhpF | + | N |  |
| 26 | YfiC | + | N |  |
| 27 | AcrR | + | N |  |
| 28 | RutR | + | + | 73.1 ± 0.1 |
| 29 | DnaX | + | N |  |
