# Supplementary material for: N ε−Lysine Acetylation of a Bacterial Transcription Factor Inhibits Its DNA-Binding Activity
Source: PLoS One. 2010 Dec 31;5(12):e15123. doi: 10.1371/journal.pone.0015123 (PMC3013089; doi:10.1371/journal.pone.0015123)
Supplement: Table S2 — rcsB plasmids and primers. aPrimers used to introduce the amino acid substitution. Nucleotide changes are underscored. bPlasmids derived from cloning vector pBAD30 [3] for in vivo analysis. cPlasmids derived from pTEV cloning vector pKLD66 [4] for overproduction and purification of products. (DOC) [file pone.0015123.s005.doc]

**Table S2. *rcsB* plasmids and primers.**

| **Mutation** | **Primers^a^** | ***rcsB* Allele** | **pBAD^b^** | **pTEV^c^** |
| --- | --- | --- | --- | --- |
| --- | --- | *rcsB^+^* | pRCSB3 | pRCSB6 |
| K 🡢A  (AAA– GCG) | 5’-GCTGAACCGCAGTATTGCGACCATCAGTAGCCAG-3’  5’-CTGGCTACTGATGGTCGCAATACTGCGGTTCAGC-3’ | *rcsB1336* | pRCSB4 | pRCSB19 |
| K 🡢 R  (AAA– CGT) | 5’-GCTGAACCGCAGTATTCGTACCATCAGTAGCCAG-3’  5’-CTGGCTACTGATGGTACGAATACTGCGGTTCAGC-3’ | *rcsB1337* | pRCSB5 | pRCSB20 |
| K 🡢 Q  (AAA– CAG) | 5’-GCTGAACCGCAGTATTCAGACCATCAGTAGCCAG-3’  5’-CTGGCTACTGATGGTCTGAATACTGCGGTTCAGC-3’ | *rcsB1338* | pRCSB7 | pRCSB10 |
