# Supplementary material for: N ε−Lysine Acetylation of a Bacterial Transcription Factor Inhibits Its DNA-Binding Activity
Source: PLoS One. 2010 Dec 31;5(12):e15123. doi: 10.1371/journal.pone.0015123 (PMC3013089; doi:10.1371/journal.pone.0015123)
Supplement: Table S3 — E. coli K-12 strains used in this studya. aStrains contain additional mutations associated with strain VH1000 = lacI lacZ pyrE +. The VH1000 = lacI lacZ pyrE + φ(flhD-lacZ) strain [5] was a gift from R. Gourse (University of Wisconsin-Madison). The strain is derived from E. coli K-12 MG1655. (DOC) [file pone.0015123.s006.doc]

**Table S3. *E. coli* K-12 strains used in this study^a^.**

|  | **Strains used for β-galactosidase activity determination** |
| --- | --- |
| **Strain Number** | **Genotype^a,b^** |
| JE11822 | φ(*flhD-lacZ*) Δ*araC536* Δ*araBAD567* Δ*rcsB770*::*kan*^+^/pBAD30 |
| JE11823 | φ(*flhD-lacZ*) Δ*araC536* Δ*araBAD567* Δ*rcsB770*::*kan*^+^/pRCSB3 *rcsB^+^* |
| JE11824 | φ(*flhD-lacZ*) Δ*araC536* Δ*araBAD567* Δ*rcsB770*::*kan*^+^/pRCSB4 *rcsB1336* (RcsB^K180A^) |
| JE11825 | φ(*flhD-lacZ*) Δ*araC536* Δ*araBAD567* Δ*rcsB770*::*kan*^+^/pRCSB5 *rcsB1337* (RcsB^K180R^) |
| JE11826 | φ(*flhD-lacZ*) Δ*araC536* Δ*araBAD567* Δ*rcsB770*::*kan*^+^/pRCSB7 *rcsB1338* (RcsB^K180Q^) |
|  | **Strains used for swimming motility assays – investigation of Lys180 substitutions** |
| **Strain Number** | **Genotype^b^** |
| JE12017 | Δ*araC536* Δ*araBAD567* Δ*rcsB770*::*kan*^+^/ pBAD30 |
| JE12018 | Δ*araC536* Δ*araBAD567* Δ*rcsB770*::*kan*^+^/ pRCSB3 *rcsB^+^* |
| JE12019 | Δ*araC536* Δ*araBAD567* Δ*rcsB770*::*kan*^+^/ pRCSB4 *rcsB1336* (RcsB^K180A^) |
| JE12020 | Δ*araC536* Δ*araBAD567* Δ*rcsB770*::*kan*^+^/ pRCSB5 *rcsB1337* (RcsB^K180R^) |
| JE12021 | Δ*araC536* Δ*araBAD567* Δ*rcsB770*::*kan*^+^/ pRCSB7 *rcsB1338* (RcsB^K180Q^) |
